# Travel-Related Cutaneous Myiasis: A Case Report

**DOI:** 10.3390/jcm13175190

**Published:** 2024-09-01

**Authors:** Alessa Z. Mendoza, Sahar Borna, Olivia A. Ho, James C. Waldorf

**Affiliations:** Division of Plastic Surgery, Mayo Clinic, 4500 San Pablo Rd., Jacksonville, FL 32224, USA

**Keywords:** *Dermatobia hominis*, myiasis, cutaneous myiasis, furuncular myiasis, human botfly

## Abstract

Background: Human myiasis, the infestation of tissues by dipterous larvae, commonly manifests as painful, raised skin lesions, particularly in tropical regions such as Latin America, where *Dermatobia hominis* (the human botfly) is a frequent cause. With increased international travel, cases of travel-related myiasis have become more prevalent, necessitating awareness among healthcare providers in non-endemic regions. Methods: We present a case of cutaneous myiasis in a 69-year-old male who returned to the United States from Belize. The patient exhibited a painful skin lesion on his right knee, initially suspected to be an insect bite. The diagnosis was confirmed through ultrasound imaging, which revealed the presence of a subcutaneous larva. Conservative larva removal efforts failed, leading to surgical extraction of the larva. Results: The surgical intervention successfully removed the larva in its entirety, with the wound healing well after the procedure. Pathological examination confirmed the larva as *Dermatobia hominis*. The patient experienced a satisfactory recovery, with no complications observed during follow-up. Conclusions: This case underscores the importance of considering myiasis in the differential diagnosis of patients presenting with painful skin lesions following travel to tropical regions. As globalization increases, healthcare providers should be vigilant in recognizing and appropriately treating travel-related diseases such as myiasis to ensure prompt and effective care.

## 1. Introduction

As COVID-19 restrictions and public concerns diminish, there have been strong rebounds in cross-border flow and international travel, meaning another potential period of intensified globalization of infectious diseases [1]. Most commonly, people acquire travel-related diseases that are gastrointestinal, dermatologic, systemic, or respiratory in nature [2].

Several Diptera families, such as Calliphoridae, Sarcophagidae, and Oestridae, are responsible for human myiasis. The classification of myiasis is based on the affected area, leading to categories such as cutaneous, urogenital, and pulmonary myiasis. The cutaneous form is the most prevalent, with *Dermatobia hominis*, the human botfly, being a frequent cause of furuncular cutaneous myiasis [3] Furuncular myiasis is characterized by the development of a disproportionately painful, boil-like inflammatory nodule with a central punctum on healthy skin, often following a history of insect bites [4].

In North America, cutaneous myiasis is most commonly caused by infestation with the larvae of *Cuterebra* [5]. However, in travelers to tropical regions, particularly in Central and South America, cutaneous myiasis is typically caused by *Dermatobia hominis*, the human botfly [3,6]. Treatment involves the removal of the larvae and sometimes the use of antibiotics [4,7].

With the recent rises in international travel and globalization, we may expect to see more such cases of myiasis and other travel-related diseases here at home.

This report offers a review of a case of cutaneous myiasis caused by *Dermatobia hominis*, or human botfly infestation, in a 69-year-old male patient who had recently returned to the United States from a trip to Latin America. Included are the details of the presentation, diagnosis, and treatment plan so that future cases of human myiasis in recent travelers can be more readily recognized, understood, and resolved.

## 2. Case Presentation

A 69-year-old male presented to the Division of Plastic Surgery of Mayo Clinic in Jacksonville, Florida, as per referral by his primary care provider for discomfort in the lateral side of his right knee. The patient reported a raised, irritated skin area, which he believed was caused by an insect bite. The lesion had serosanguinous drainage without pus. Despite the skin manifestation, he was asymptomatic and denied any similar lesions in the past. He also denied experiencing fevers, chills, fatigue, or any other symptoms besides skin irritation. He was treating it with Neosporin and triple antibiotic ointment.

The patient had traveled to Belize approximately six weeks before the visit. While walking in the jungle, he recalled being stung by an insect. Shortly afterward, he noticed a skin lesion on the lateral side of his right knee. He was then seen by his primary care physician and prescribed doxycycline 100 mg to be taken orally twice a day for 7 days and mupirocin 2% topical antibiotic to be applied three times daily. After complying with treatment for 4 days, he elected to obtain an ultrasound and to see other specialists due to a lack of significant improvement.

**Figure 1:**
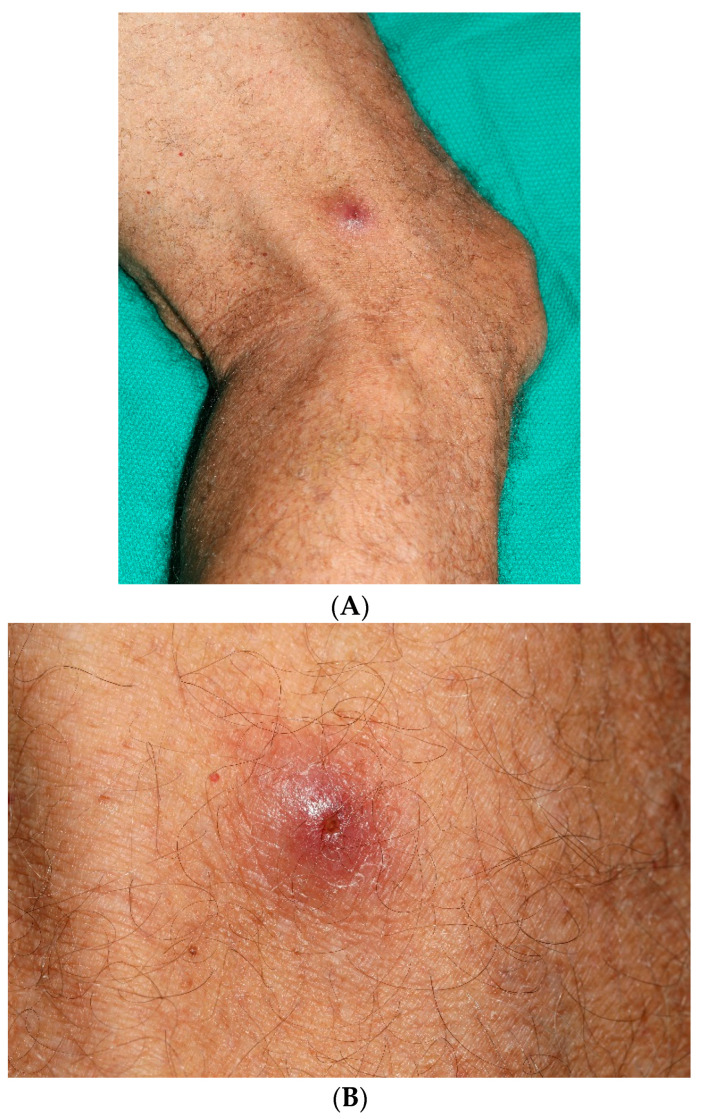
(**A**) Elevated nodule with a central punctum observed on the lateral side of the patient’s right thigh. (**B**) Closer view highlighting the punctum and surrounding erythema.

Real-time grayscale ultrasound supplemented by color and spectral Doppler ultrasound was performed on the lateral side of the patient’s right leg. The ultrasound examination revealed subcutaneous edema and associated skin swelling and thickening at the palpable site of concern. Heterogeneous, ill-defined fluid collection was observed surrounding a central echogenic 6 mm foreign body with shadowing and punctate echogenic foci. The surrounding fluid component measured up to 2 cm. The central ovoid foreign body exhibited spontaneous motion in multiple cine clips, with an associated color Doppler artifact. A linear tract extended from the foreign body to the skin, with an opening at the skin surface. Periodic motion within the skin tract was noted on continuous observation, which raised suspicion for a subcutaneous botfly larva. Following the ultrasound, he visited Mayo Clinic Plastic Surgery, and upon physical examination, the physician noted an elevated nodule with a central punctum on the lateral side of the patient’s right thigh, as seen in Figure 1A,B. The patient’s history of recent travel to a tropical region, imaging results, and physical examination findings seemed consistent with cutaneous myiasis.

**Figure 2:**
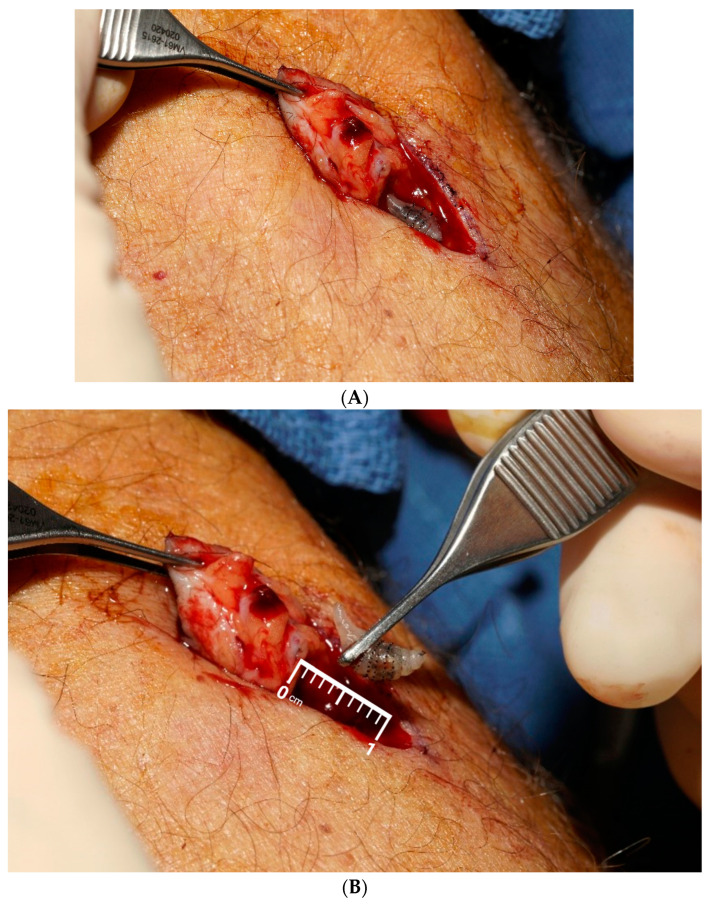
(**A**) Elliptical excision of the entire cutaneous manifestation, with dissection extending down to the underlying deep fascia. (**B**) Live larva encountered and removed in its entirety, remaining viable upon extraction.

In the office, multiple conservative measures were taken to force the larva to surface, but all were unsuccessful. After a discussion of treatment alternatives, the patient opted to proceed with surgical intervention. Following routine preparation, the area of concern was infiltrated with 1% xylocaine with epinephrine. The skin opening was enlarged and filled with betadine. Despite these efforts, the larva could not be brought to the surface. It was decided to proceed with total excision of the area. An elliptical excision was performed, including the entire cutaneous manifestation, with dissection extending down to the underlying deep fascia. A live larva was encountered and removed in its entirety, remaining viable upon extraction, Figure 2A,B. A thorough inspection of the wound revealed no residual material. The wound was then cleaned with betadine solution and closed using interrupted 4-0 Vicryl for the deep tissue and interrupted 4-0 Prolene in a horizontal mattress stitch for the skin. The patient was placed on antibiotics, which were continued until completion. A follow-up appointment was scheduled in 10 days for suture removal, with instructions to contact the clinic if any issues arose. The larva, measuring 1.0 cm and pale tan in color, was sent to pathology for identification. A pathologist diagnosed it as “*Dermatobia hominis*”, commonly known as the human botfly maggot, using digital imaging and visual inspection. The pathology examination showed benign skin and subcutaneous tissues with focal necrotizing granulomatous inflammation and a giant cell reaction.

The patient returned a week after the extraction procedure for a follow-up examination and suture removal. He appeared to be healing well with no complications and stated that he was satisfied with his treatment.

## 3. Discussion

Dermatological diseases affect up to 8% of travelers, with cutaneous larval parasites being a top cause [8]. Out of these, myiasis represents 3.5–9.3% of dermatological conditions seen in patients who have recently traveled to the tropics [9].

Myiasis is a parasitic, cutaneous infestation that is caused by the larvae of dipterous flies and typically manifests as painful, punctate nodules [6,10]. Furuncular lesions result when the dipterous fly deposits its eggs on a mosquito or other flying insect, which then lands on human skin where the eggs will hatch, penetrate a site, and then develop subcutaneously for up to 14 weeks [11]. In Latin America, *Dermatobia hominis* is the main cause of human myiasis, and it rarely occurs outside of this region except with visitors returning to their home countries [9].

Soon, however, *Dermatobia hominis* myiasis will likely gain greater relevance outside of Latin America. This is expected due to the easing of restrictions on travel, and there is the potential to see more cases involving travel-related diseases such as myiasis [1,12]. Thus, proper differential diagnosis of *Dermatobia hominis* myiasis and understanding of how to treat this disease is increasingly imperative for physicians in primary care, dermatology, plastic surgery, and related specialties in quickly and accurately handling the needs of patients returning from their international trips.

Diagnosing myiasis is clinical and relies on both physical examination and history [13,14]. As previously described, myiasis causes symptoms of painful nodules, usually with central puncta, as well as pain, nocturnal pruritus, or general discomfort in the skin surrounding the site [6,10,11,15]. Patients may also have the sensation of crawling under their skin, and some even report systemic symptoms such as sweating and nausea [13,15]. Myiasis typically affects exposed, accessible areas where flying insects can deposit the dipterous fly eggs, such as the skin on an individual’s limbs or neck [16]. A case has also been reported of a myiasis-induced mass in the periauricular area of a child [17]. This is in keeping with our case, as the patient experienced cutaneous myiasis on the lateral aspect of his right knee. Myiasis has been reported in less exposed areas as well, such as the inguinal and genital areas, the breasts, and the scalp [16,18,19,20,21]. The presentations of *Dermatobia hominis* infestations show considerable variation. Similar to our patient, a 48-year-old woman who had traveled to Belize had lesions on her left ankle and leg, presenting two weeks after travel [22]. Similarly, a 63-year-old woman returning from Belize had an initially painless shoulder swelling that became painful and exudative [23]. Another traveler to Belize developed a lesion on his left upper eyelid with a central pore and discharge [24]. In contrast, a 22-year-old woman from Costa Rica had a pruritic, painless forearm lesion, while a 31-year-old man who had also visited Belize had tender, erythematous boils on his abdomen [23]. A 59-year-old from Mexico and Guatemala and a 29-year-old from Taiwan presented with erythematous lesions with central pores but differed in associated symptoms such as pain and lymphadenopathy [25,26]. With such wide variation of myiasis nodule sites, the general nature of myiasis symptoms and the long incubation period of the larva can complicate diagnosis and have led to many instances of initial misdiagnosis of the nodules as anything from breast cancer to a simple trauma-related lesion and leishmaniasis [7,11,16,21].

In this case, the patient had a delayed correct diagnosis and management, given that he attributed his area of cutaneous myiasis to an insect bite. His primary care team subsequently treated it as an infected bite or wound with antibiotics. This patient was then fortunate enough to receive radiologic imaging and assessment quickly from our plastic surgery team. However, others may face prolonged misdiagnosis and be treated with incorrect intervention. Physicians must take careful patient histories and consider the possibility of myiasis in patients who have traveled to the tropics and present with painful nodules or lesions, whether on those commonly exposed areas or not.

Upon suspicion of myiasis, diagnosis can then be made through imaging and identification of the larva by pathology or entomology following removal. Ultrasound has been deemed very useful in previous cases, as well as the one reported herein, where it was used to confirm suspicions of myiasis and visualize the larva before attempts at extraction [18]. Other forms of imaging, such as magnetic resonance imaging or computed tomography, may also be useful when it comes to anatomical sites where myiasis is less common [16]. Atypical presentations and locations may require imaging the most [16]. For instance, in one patient, botfly myiasis presented as dacryocystitis, which was diagnosed using a contrast-enhanced CT scan of the sinuses [5]. In another case featuring a periauricular mass, a CT scan, MRI, and magnetic resonance angiography (MRA) were used to diagnose the myiasis and assess the vascular involvement [17]. Several similarities and differences emerge in comparing the diagnosis of cutaneous lesions across various cases. For instance, erythematous lesions with central pores were a common feature in multiple cases, such as the 1.2 cm lesion on the scalp and the 15 mm nodules on the abdomen, which were initially suspected to be leishmaniasis but later identified as myiasis upon biopsy and larval extraction [23,26]. Additionally, both cases involving diffuse subcutaneous edema and sinus tracts, as seen in the MRI-diagnosed lesions on the ankle and the need for neuroimaging in a child suspected of calvarial osteomyelitis, highlight the challenges in differentiating among various parasitic and infectious etiologies [22,27]. A notable difference is that while some cases presented with clear central pores indicating myiasis, others required more invasive procedures such as incision or ultrasound to confirm the presence of larvae and ensure complete removal [23,25]. The variability in presentation—from ulcerative dermatitis to granulomatous reactions—emphasizes the importance of a comprehensive diagnostic approach, combining clinical examination, imaging, and histopathology to accurately differentiate between conditions such as leishmaniasis and myiasis [23,26].

Once the physician is quite certain that a nodule has resulted from myiasis, treatment is mostly straightforward. The aim of treatment is to completely extract the larva with minimal trauma to surrounding tissue. Noninvasive methods are considered first and range from the use of a venom extractor along with pressure-increasing lidocaine injections to simple occlusive therapies, such as the application of petroleum jelly, clear nail polish, or solid fats to the central pore to promote the emergence of any larvae to the surface [9,28]. A similar case involving a lateral arm lesion caused by myiasis was diagnosed in the emergency department and successfully treated by occluding the larva’s air vent with petroleum jelly and a Tegaderm™, which led to the larva exiting the lesion [15]. If these more conservative efforts fail, the next option to consider is surgical removal via elliptical or punch excision [16,28]. In some cases, deeper incisions were necessary to remove large larvae, such as those measuring 13 mm and 11 mm [22] or a 2 cm larva removed under local anesthetic [23]. Other treatments included coal tar ointment, which led to larval expulsion after clindamycin failed [25], and in some instances, a deeper incision was required to reveal the larva despite initial treatments with antibiotics and superglue [22]. Similarly, erythromycin treatment led to lesion exudation and larval visibility through a central pore before successful removal [23]. Additional methods included using forceps for gentle extraction and morphological diagnosis in one case [26] and visual inspection following excision in another case [24]. With this patient, initial attempts were made to use less invasive measures for removal in the form of a small puncture and betadine injection for pressure increase, but these attempts proved insufficient for total extraction. This had been anticipated as a possible issue; accordingly, we instead moved forward with total elliptical excision of the area followed by routine wound debridement and closure. Preparations for total excision in case of noninvasive removal failure were imperative to our treatment plan’s success.

As seen in our case, there is usually only one larva [14,15], and regardless of the extraction method, complete removal of the larva with minimal damage or secondary infection risk to surrounding tissue should allow the skin to heal well, as observed with the patient in this case report.

## 4. Conclusions

This case report details the presentation, diagnosis, and treatment of cutaneous myiasis caused by Dermatobia hominis in a patient who acquired the infection during travel to Belize. The findings emphasize the increasing relevance of travel-related diseases in our globally connected world. It is crucial for physicians to obtain thorough travel histories and be vigilant for such infections in patients returning from endemic areas. This report’s limitations include its focus on a single case, which may not generalize to all instances of cutaneous myiasis, and its brief follow-up period, which limits insights into long-term outcomes. Future research should aim to include a broader range of cases to validate treatment approaches, compare different treatment modalities, and refine diagnostic techniques. Enhanced physician awareness and education regarding travel-related diseases will also be essential in improving early detection and management.

## Data Availability

The original contributions presented in the study are included in the article, further inquiries can be directed to the corresponding author.

## References

[1] Altman S.A., Bastian C.R. (2022). The State of Globalization in 2022. https://hbr.org/2022/04/the-state-of-globalization-in-2022.

[2] Chen L.H., Blair B.M. (2015). Infectious Risks of Traveling Abroad. Microbiol. Spectr..

[3] Papineni V., Dieu S., Rennie W.J. (2023). The human botfly “bubbling sign”: Ultrasound features of cutaneous furuncular myiasis. Indian J. Radiol. Imaging.

[4] Ragi S.D., Kapila R., Schwartz R.A. (2021). The botfly, a tropical menace: A distinctive myiasis caused by Dermatobia hominis. Am. J. Clin. Dermatol..

[5] Rozanski C.A., DeSimone J.D., Milman T., Ramesh S. (2023). Botfly Myiasis Masquerading as Dacryocystitis. Ophthalmic Plast. Reconstr. Surg..

[6] Francesconi F., Lupi O., Tyring S.K., Lupi O., Hengge U.R. (2017). 31-Myiasis. Tropical Dermatology.

[7] Jallow B.J., Gassara G., Bajinka O., Luo Y., Liu M., Cai J., Huang J., Meng F. (2024). Human myiasis in Sub-Saharan Africa: A systematic review. PLoS Neglected Trop. Dis..

[8] Fitzpatrick J.E., High W.A., Kyle W.L., Fitzpatrick J.E., High W.A., Kyle W.L. (2018). Chapter 36-Cutaneous Diseases of Travelers. Urgent Care Dermatology: Symptom-Based Diagnosis.

[9] Zammarchi L., Viligiardi R., Strohmeyer M., Bartoloni A. (2014). Dermatobia hominis: Small Migrants Hidden in Your Skin. Ann. Dermatol..

[10] Showler A.J., Wilson M.E., Kain K.C., Boggild A.K. (2014). Parasitic diseases in travelers: A focus on therapy. Expert Rev. Anti. Infect. Ther..

[11] Tamir J., Haik J., Orenstein A., Schwartz E. (2003). Dermatobia hominis myiasis among travelers returning from South America. J. Am. Acad. Dermatol..

[12] Torner N. (2023). The end of COVID-19 public health emergency of international concern (PHEIC): And now what?. Vacunas (Engl. Ed.).

[13] Mattern J.Q., Barbul A. (2003). Human botfly. Am. J. Surg..

[14] Velev V., Dinkova M., Mirtschew A. (2019). Human botfly infection. QJM Int. J. Med..

[15] Mahal J.J., Sperling J.D. (2012). Furuncular myiasis from Dermatobia hominus: A case of human botfly infestation. J. Emerg. Med..

[16] Shenouda M., Enten G., Nguyen T., Mangar D., Camporesi E. (2018). Human Botfly: A Case Report and Overview of Differential Diagnosis. J. Investig. Med. High Impact Case Rep..

[17] Boruk M., Rosenfeld R.M., Alexis R. (2006). Human botfly infestation presenting as peri-auricular mass. Int. J. Pediatr. Otorhinolaryngol..

[18] Jones C.H., Leon M., Auerbach J., Portillo-Romero J. (2020). Ultrasound Detection of Human Botfly Myiasis of the Scalp: A Case Report. Cureus.

[19] Dunphy L., Sood V. (2019). The human botfly’ presenting as a scalp lesion. BMJ Case Rep..

[20] Gaci R., Delord M., Parola P., Brouqui P., Lagier J.C. (2015). Extended Perineal Dermatobia hominis Myiasis in a Traveler Returning From South America. JAMA Dermatol..

[21] Kahn D.G. (1999). Myiasis secondary to Sermatobia hominis (human botfly) presenting as a long-standing breast mass. Arch. Pathol. Lab. Med..

[22] Cottom J.M., Hyer C.F., Lee T.H. (2008). Dermatobia hominis (Botfly) Infestation of the Lower Extremity: A Case Report. J. Foot Ankle Surg..

[23] Pallai L., Hodge J., Fishman S.J., Millikan L.E., Phelps R.G. (1992). Case Report: Myiasis—The Botfly Boil. Am. J. Med. Sci..

[24] Ofordeme K.G., Papa L., Brennan D.F. (2007). Botfly myiasis: A case report. Can. J. Emerg. Med..

[25] Maier H., Hönigsmann H. (2004). Furuncular myiasis caused by Dermatobia hominis, the human botfly. J. Am. Acad. Dermatol..

[26] Hu J.-M., Wang C.C., Chao L.L., Lee C.S., Shih C.M. (2013). First report of furuncular myiasis caused by the larva of botfly, Dermatobia hominis, in a Taiwanese traveler. Asian Pac. J. Trop. Biomed..

[27] Vijay K., Kalapos P., Makkar A., Engbrecht B., Agarwal A. (2013). Human botfly (*Dermatobia hominis*) larva in a child’s scalp mimicking osteomyelitis. Emerg. Radiol..

[28] Diaz J.H. (2006). The epidemiology, diagnosis, management, and prevention of ectoparasitic diseases in travelers. J. Travel. Med..

